# Markers of T Cell Infiltration and Function Associate with Favorable Outcome in Vascularized High-Grade Serous Ovarian Carcinoma

**DOI:** 10.1371/journal.pone.0082406

**Published:** 2013-12-23

**Authors:** Katelin N. Townsend, Jaeline E. Spowart, Hassan Huwait, Sima Eshragh, Nathan R. West, Mary A. Elrick, Steve E. Kalloger, Michael Anglesio, Peter H. Watson, David G. Huntsman, Julian J. Lum

**Affiliations:** 1 Trev and Joyce Deeley Research Centre, British Columbia Cancer Agency, Victoria, British Columbia, Canada; 2 Department of Biochemistry and Microbiology, University of Victoria, Victoria, British Columbia, Canada; 3 Anatomical Pathology, Vancouver General Hospital, Vancouver, British Columbia, Canada; 4 Department of Pathology and Laboratory Medicine, University of British Columbia, Vancouver, British Columbia, Canada; 5 Department of Biology, University of Victoria, Victoria, British Columbia, Canada; 6 Centre for the Translational & Applied Genomics, British Columbia Cancer Agency, Vancouver, British Columbia, Canada; Philipps University, Germany

## Abstract

**Background:**

When T cells infiltrate the tumor environment they encounter a myriad of metabolic stressors including hypoxia. Overcoming the limitations imposed by an inadequate tumor vasculature that contributes to these stressors may be a crucial step to immune cells mounting an effective anti-tumor response. We sought to determine whether the functional capacity of tumor infiltrating lymphocytes (TIL) could be influenced by the tumor vasculature and correlated this with survival in patients with ovarian cancer.

**Methodology and Principal Findings:**

In 196 high-grade serous ovarian tumors, we confirmed that the tumor vascularity as measured by the marker CD31 was associated with improved patient disease-specific survival. We also found that tumors positive for markers of TIL (CD8, CD4 and forkhead box P3 (FoxP3)) and T cell function (granzyme B and T-cell restricted intracellular antigen-1 (TIA-1)) correlated significantly with elevated vascularity. *In vitro*, hypoxic CD8 T cells showed reduced cytolytic activity, secreted less effector cytokines and upregulated autophagy. Survival analysis revealed that patients had a significant improvement in disease-specific survival when FoxP3 expressing cells were present in CD31-high tumors compared to patients with FoxP3 expressing cells in CD31-low tumors [HR: 2.314 (95% CI 1.049–5.106); *p* = 0.0377]. Patients with high vascular endothelial growth factor (VEGF) expressing tumors containing granzyme B positive cells had improved survival compared to patients with granzyme B positive cells in VEGF-low tumors [HR: 2.522 (95% CI 1.097–5.799); *p* = 0.0294].

**Significance:**

Overall, this data provides a rationale for developing strategies aimed at improving the adaptability and function of TIL to hypoxic tumor conditions.

## Introduction

Epithelial ovarian carcinoma is classified into five main subtypes including high-grade serous, endometrioid, clear cell, mucinous and low-grade serous [1]. Each ovarian subtype is genetically distinct and responds differently to therapy [1]. The survival rate of ovarian carcinoma patients has not changed over the past 30 years since the introduction of platinum-based chemotherapy treatment [2] and it is the most lethal of the gynecological malignancies [1]. Following cytoreductive surgery and chemotherapy tumors often recur, resulting in median overall survival for stage III/IV patients ranging from 14.6–50.9 months depending on the histological subtype [3]. The poor outcomes associated with ovarian cancer highlight the need for improved therapies and a better understanding of the disease.

One factor that has been shown to be associated with positive outcomes in ovarian cancer patients is the immune system. In particular, the presence of CD3 and CD8 tumor-infiltrating T cells is associated with a significant survival advantage, specifically in high-grade serous ovarian cancer patients [4], [5]. While other ovarian subtypes such as endometrioid and clear cell ovarian carcinoma show infiltration by CD8 T cells, their presence does not correlate with improved patient survival [4], [5]. T regulatory cells (T regs) have also been identified in various ovarian carcinoma subtypes and have been shown to be a predictor of poor survival outcomes [6], [7]. However, cells expressing FoxP3, a transcription factor associated with T regs [8], have also been observed as beneficial for ovarian cancer patient survival outcomes [5], [9]. Overall, these findings indicate that ovarian carcinomas are immunogenic, providing a relevant setting to study the immune response.

The tumor environment is an important consideration for immune activity. Tumor cell growth and proliferation place a high bioenergetic demand on the host tumor environment to provide and maintain sufficient levels of nutrients [10]. Oxygen delivery to tumors can be limited by the development of abnormal tumor micro-vessels and a decrease in oxygen diffusion, which results in tissue hypoxia. Serous ovarian tumors have been reported to exhibit features consistent with a state of hypoxia [11]. Thus, we reasoned that hypoxia can influence a T cell as it migrates into hypoxic tissue and we assessed whether this has an impact on the overall anti-tumor immune response.

In response to hypoxia, T cells stabilize the alpha subunit of the transcription factor hypoxia-inducible factor-1 (HIF-1α). This allows T cells to reprogram their metabolism to maintain cellular function under hypoxia [12]. It has been shown that T cell function including cytokine secretion is altered under hypoxia. Hypoxia can skew CD4 T cell differentiation and cause degradation of FoxP3 resulting in a concurrent increase in T helper type 17 (Th17) CD4 T cells [13]. In contrast, hypoxia has also been shown to promote the expression of FoxP3 in CD4 T cells *via* HIF-1α induction [14]. In addition, environmental hypoxia in ovarian tumors promoted the recruitment of T reg cells in another report [15]. These findings are important for anti-tumor immunity because inhibiting hypoxia could have a profound effect on T cell-mediated tumor killing [16]. This was shown in a recent study where normalizing the tumor vasculature enhanced the efficacy of a breast cancer vaccine in a mouse model [17].

In the current study, we sought to compare the survival outcomes of patients with non-vascularized high-grade serous ovarian tumors harboring immune infiltrates to patients with infiltrates in vascularized tumors. CD31 and VEGF, two established markers were chosen as proxies for tumor hypoxia and vascularity [18], [19]. We hypothesized that TIL and TIL function would be decreased in hypoxic conditions and this would be associated with a reduction in tumor control and patient outcomes. Given that approximately 80% of high-grade serous patients present at an advanced stage when tumor eradication by surgery and chemotherapy is difficult [1], understanding immune parameters may be particularly beneficial for developing future treatments in this subtype.

## Results

### Expression of CD31 in high-grade serous ovarian tumors is associated with improved patient survival

**Figure 1:**
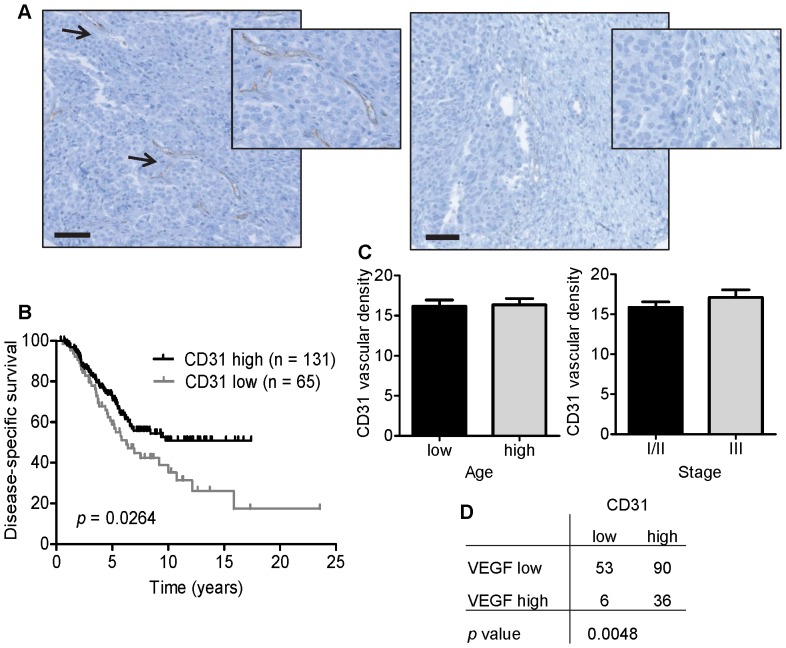
High-grade serous ovarian tumors express the vasculature marker CD31. (A) An image of high (left panel) or low (right panel) CD31 immunohistochemistry staining scanned at 10× magnification. Scale bar = 50 µm. Inset images show high (left panel) or low (right panel) CD31 staining when scanned at 40× magnification. Arrowheads indicate brown regions of CD31 staining (B) Kaplan-Meier analysis of disease-specific survival in high-grade serous ovarian cancer patients. Statistical significance was assessed using a log-rank test. (C) Mean CD31 vascular density comparisons with patient age (split on the median) and disease stage (*p* = not significant). Statistical significance was assessed using a Mann Whitney rank-sum test. (D) Contingency analysis of CD31 and VEGF. Statistical significance was assessed by Fisher's exact test.

We examined CD31 expression in 196 high-grade serous ovarian tumor patients (Table 1). CD31 is highly expressed on endothelial cells and is a well-established blood vessel marker for assessing the extent of angiogenesis [20]. The expression of CD31 in tumors is one indication of vascularization and an indirect measurement of an adequate oxygen supply. In animal models, poor vascularization leads to limitations in oxygen supply, resulting in hypoxia [21], [22]. Shown in Figure 1A is a representative tumor core from a patient with a typical pattern of high CD31 staining (left panel) and a tumor core from a patient with low levels of CD31 staining (right panel). In this cohort, patients with higher levels of CD31 staining had significantly improved disease-specific survival compared to patients with lower CD31 staining [HR: 1.657 (95% CI 1.061–2.588); *p* = 0.0264] (Figure 1B). There was also a modest increase towards improved overall survival and progression-free survival in CD31-high patients (Figure S1). Clinical factors such as patient age or stage did not correlate with CD31 levels (Figure 1C). The tissue cores were also stained for VEGF, an angiogenic factor which was strongly correlated with CD31 (*p* = 0.0048, Figure 1D) [18]. Based on this analysis and the absence of a direct hypoxia marker such as pimonidazole, we used both CD31 and VEGF as markers to define the extent of tumor hypoxia.

**Table 1:** Patient characteristics, follow-up time and survival characteristics for high-grade serous ovarian carcinoma cases.

**Age at surgery (years)**	
Median	60.6
Range	37.6–86.0
**Silverberg grade**	
1	0 (0%)
2	54 (27.6%)
3	141 (71.9%)
Not graded	1 (0.5%)
**FIGO stage** 1	
I	47 (24.0%)
II	84 (42.9%)
III	65 (33.2%)
Total number patients	196
**Follow-up time**	
Median follow-up (range), years	5.3 (0.4–23.6)
**Survival characteristics**	
Disease progressions	100 (51.0%)
Ovarian cancer deaths	90 (45.9%)
Total number of deaths	120 (61.2%)

1 FIGO = Federation of Gynecology and Obstetrics.

### Highly vascularized tumors are positive for TIL markers

**Figure 2:**
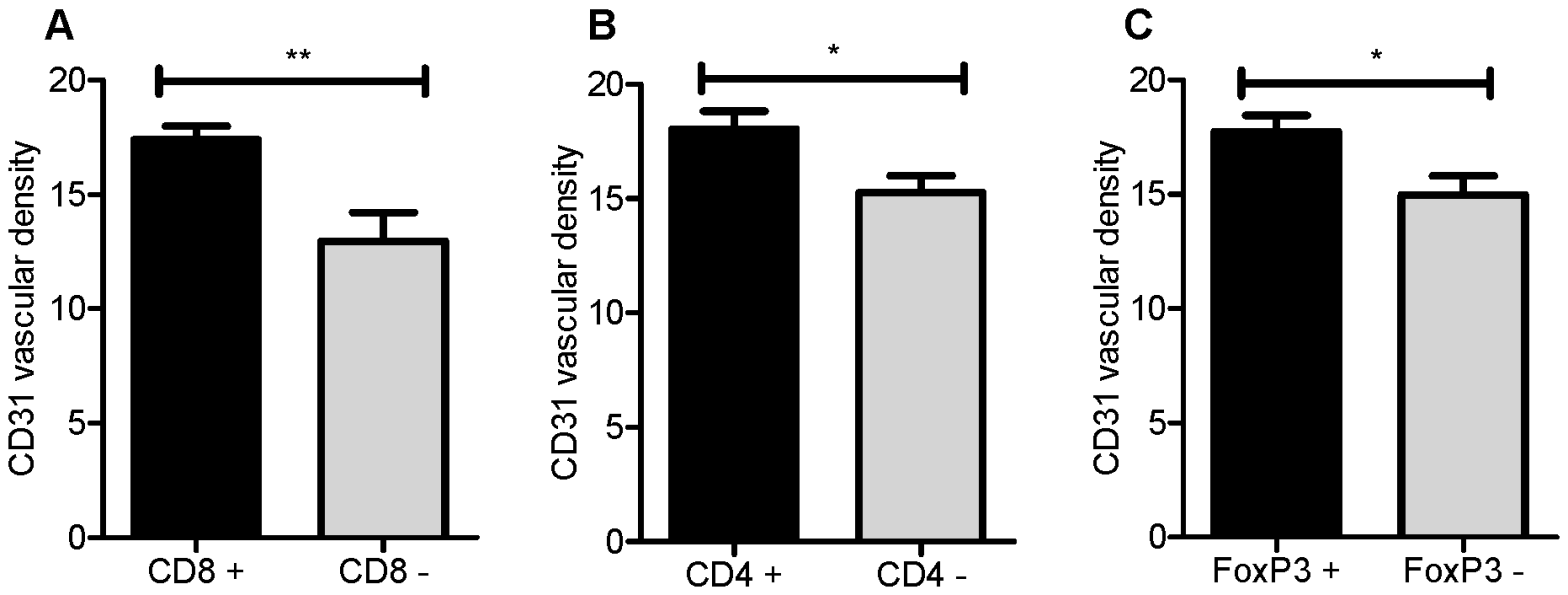
High-grade serous tumors containing TIL have higher vascular density scores than those without TIL. Mean CD31 vascular density scores were compared in relation to expression of (A) CD8, (B) CD4 and (C) FoxP3. Error bars indicate standard error of the mean. Statistical significance was assessed by Mann Whitney test. *p<0.05, ** *p*<0.01.

To determine whether tumors with TIL present were more likely to express CD31, we enumerated the mean vascular density score of CD31 in patients who had CD8, CD4 or FoxP3 expression within their tumors (Figure 2). Patients were separated into two groups corresponding to those containing T cells within their tumors and those lacking T cells. In this analysis, tumors positive for immune cell markers had higher mean vascular density scores than tumors negative for immune cell markers. This relationship was significant for all markers analyzed: CD8 (*p* = 0.0017, Figure 2A), CD4 (*p* = 0.0112, Figure 2B) and FoxP3 (*p* = 0.0147, Figure 2C). The positive relationship between immune cell infiltration and tumor vascularization was also observed by contingency analysis (Table S1). High VEGF expression was similarly associated with CD4 (*p* = 0.0002) or FoxP3 (*p* = 0.0052) positive cells (Table S1), whereas the relationship was not significant with CD8 cells (*p* = 0.2078).

### Tumors containing the functional immune markers granzyme B and TIA-1 are highly vascularized

**Figure 3:**
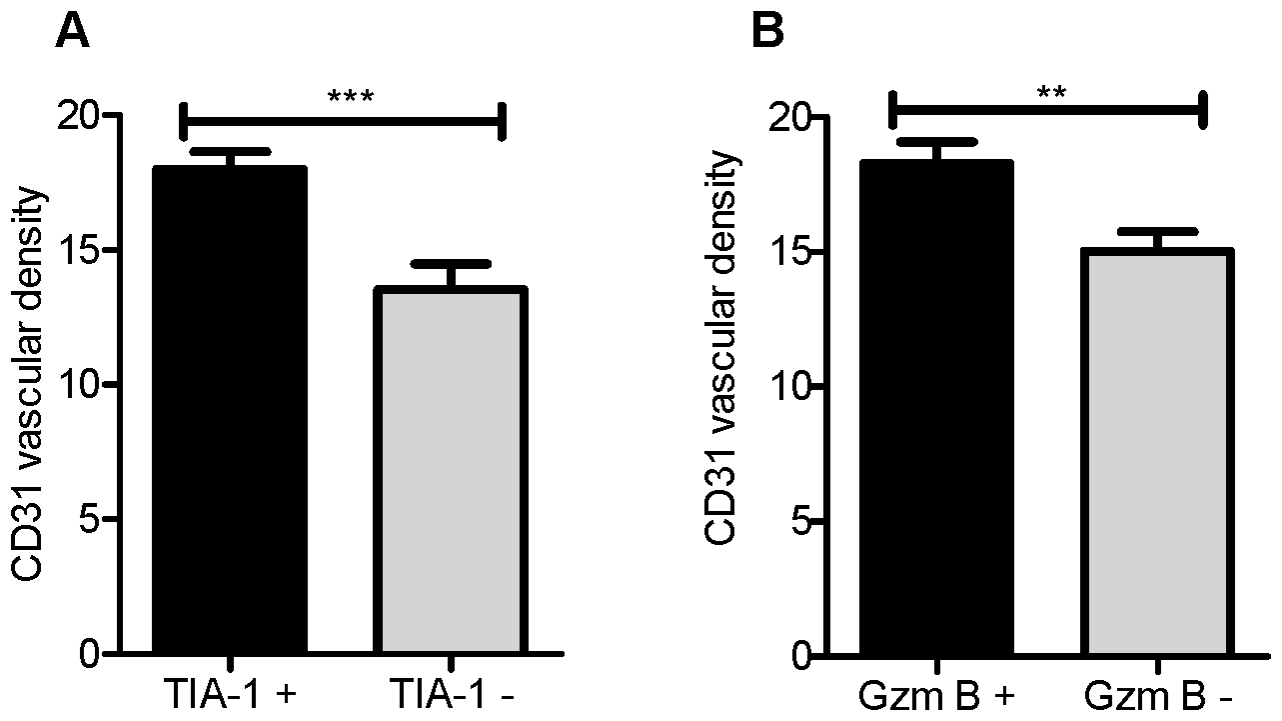
High-grade serous tumors containing markers of immune function have higher vascular density scores. Mean CD31 vascular density scores were compared in relation to the expression of (A) TIA-1 and (B) granzyme B (GzmB) tumor infiltrates in high-grade serous tumors. Error bars indicate standard error of the mean. Statistical significance was assessed by Mann Whitney test. ** *p*<0.01, ***p<0.001.

The differences in T cell infiltration and levels of tumor vascularization between tumors prompted us to examine the levels of CD31 in tumors which also expressed markers of T cell function. TIA-1 and granzyme B are markers of cytotoxicity [23], [24]. Both TIA-1 and granzyme B were associated with high CD31 vascular density scores (*p* = 0.0003 and *p* = 0.0040, Figure 3A and Figure 3B respectively). These observations were also seen when a contingency analysis was applied to the dataset (Table S1).

### Hypoxia suppresses CD8 T cell effector function

**Figure 4:**
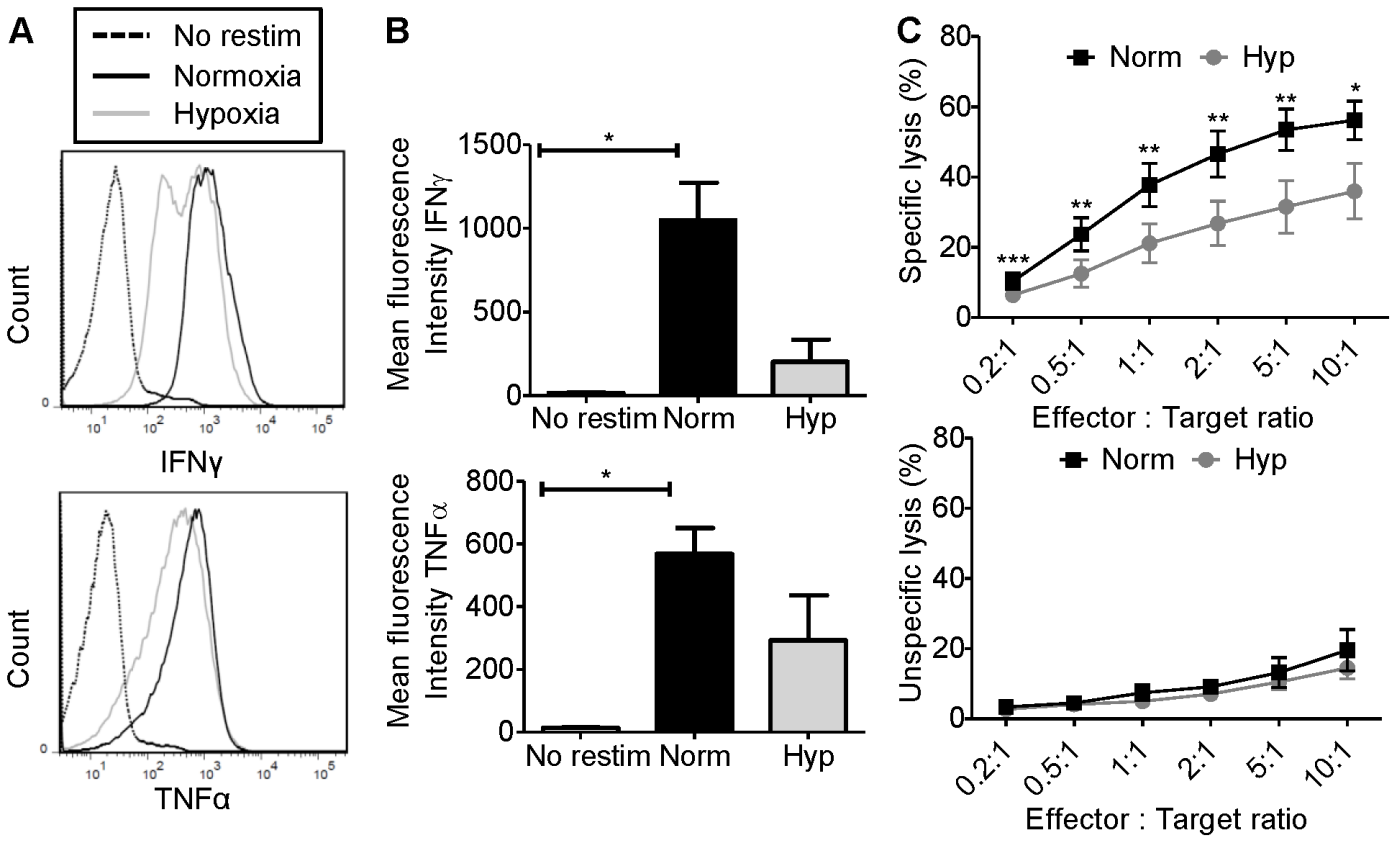
Hypoxia suppresses CD8 T cell effector function. (A) Representative histogram plots for the mean fluorescence intensity of IFNγ (top panel) and TNFα (bottom panel) produced by CD8 T cells cultured under normoxic conditions or 1.5% oxygen. (B) The average geometric mean fluorescence and standard error of the mean for 3 independent experiments is shown. Statistical significance was determined by paired t-tests. (C) OT-I T cells were co-cultured with E.G7 (top panel) or EL4 (bottom panel) target cells at various effector∶target ratios for 4 hours under normoxia (norm) or 1.5% oxygen (hyp). The mean and standard error of the mean of 4 or 5 independent cytotoxicity experiments is shown. Statistical significance was determined by paired t-tests on log transformed values. *p<0.05, ** *p*<0.01, ***p<0.001.

To determine the impact of hypoxia on CD8 T cell function, we assessed cytokine production by cytotoxic T cells. A concentration of 1.5% oxygen was chosen to represent hypoxia as this has been reported to be within the oxygen range found in tumors [25]. For this investigation, we used the murine OT-I system to assess immune activity against a known antigen [26]. OT-I splenocytes were stimulated with the SIINFEKL peptide from the ovalbumin (OVA) protein for 6 days followed by culture under 21% oxygen (normoxia) or 1.5% oxygen (hypoxia) for 15 hours. Cultures were then restimulated or not under normoxia or hypoxia for an additional 5 hours and the expression of tumor necrosis factor α (TNFα) and interferon γ (IFNγ) were quantified by intracellular flow cytometry (Figure 4A). OT-I T cells cultured under hypoxia trended toward production of less IFNγ (*p* = 0.0627) and showed a modest decrease in TNFα (*p* = 0.2819) compared to T cells cultured under normoxia (Figure 4B). We further assessed T cell function under low oxygen by measuring the ability of OT-I T cells to kill OVA expressing E.G7 target cells or OVA-negative parental EL4 control cells. OT-I cells cultured under 1.5% oxygen with E.G7 tumor targets showed a dramatic reduction in cytotoxic killing activity when compared to OT-I cells cultured at 21% oxygen. This suppression of killing activity by hypoxia was observed at all effector-to-target ratios: 0.2∶1 (*p* = 0.0002), 0.5∶1 (*p* = 0.0032), 1∶1 (*p* = 0.0055), 2∶1 (*p* = 0.0019), 5∶1 (*p* = 0.0086) and 10∶1 (*p* = 0.0127, Figure 4C). OT-I cells cultured with control EL4 cells displayed minimal lytic activity regardless of oxygen levels.

### Hypoxia induces autophagy in CD8 T cells

**Figure 5:**
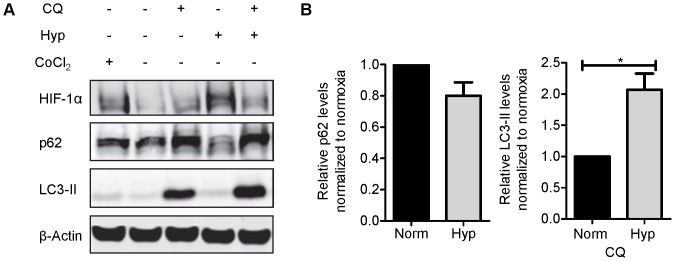
Hypoxia activates autophagy in CD8 T cells. (A) A representative Western blot indicating protein expression of HIF-1α, p62, LC3-II and β-actin is shown for OT-I CD8 T cells cultured under 1.5% oxygen. Cobalt chloride (CoCl_2_) treatment was used as a positive control for HIF-1α protein expression. (B) Autophagy induction is shown by quantification of decreasing p62 levels without chloroquine (CQ) treatment and (C) LC3-II accumulation with CQ treatment under normoxia (norm) or 1.5% oxygen (hyp). The mean and standard error of the mean for the fold change of 3 independent experiments is reported. Statistical significance was determined using a one-sample t-test on log transformed values compared to a hypothetical mean of zero. *p<0.05.

Next we sought to evaluate how CD8 T cells might adapt to the reduction in tumor vasculature. As expected, cells cultured at 1.5% oxygen for 20 hours stabilized the transcription factor HIF-1α (Figure 5A). As a potential survival mechanism [27], T cells exhibited a dramatic increase in the activation of autophagy while under hypoxic conditions. In contrast to T cells cultured under normoxia, an autophagy flux assay demonstrated an increased accumulation of the autophagy marker LC3-II under hypoxia (Figure 5A, B; lane 5 versus lane 3). In addition, hypoxic T cells showed decreased levels of the autophagy substrate p62 as compared to cells cultured under normoxia (Figure 5A, B; lane 4 versus lane 2). Together, these results demonstrate the induction of autophagy in CD8 T cells under hypoxic conditions.

### TIL and markers of immune function in poorly-vascularized tumors correlate with decreased patient survival outcomes

**Figure 6:**
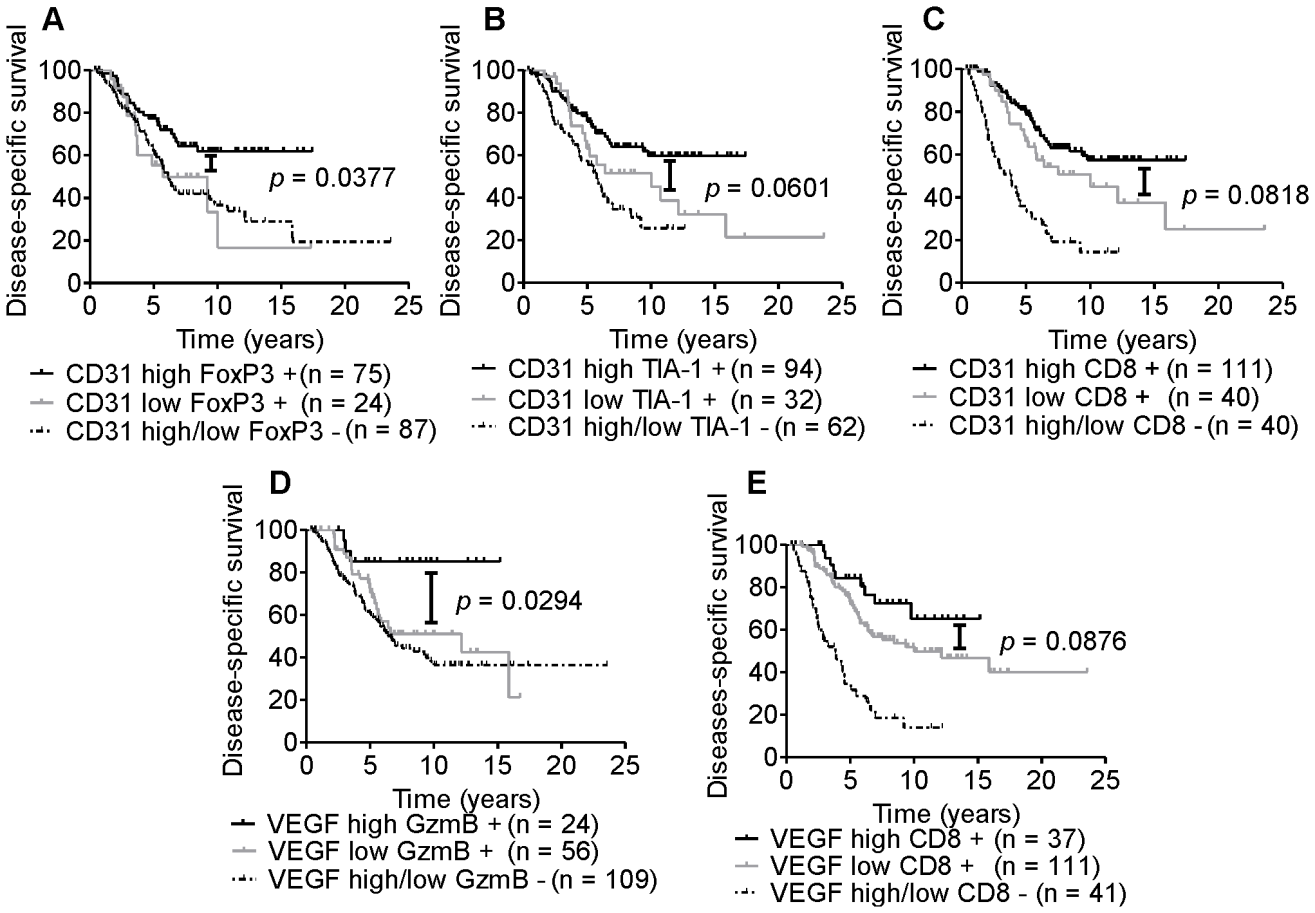
TIL and markers of immune function are associated with improved patient outcome. Kaplan-Meier analysis of disease-specific survival of high-grade serous ovarian carcinoma patients categorized by CD31, VEGF or the indicated immune markers: (A) CD31 and FoxP3, (B) CD31 and TIA-1, (C) CD31 and CD8, (D) VEGF and Granzyme B (GzmB), (E) VEGF and CD8. The statistical significance was determined using a log-rank test.

To determine if the prognostic relevance of TIL depends on tumor vascularity, patients were separated into TIL positive or negative categories. Tumors positive for TIL were further separated into two groups based on low or high vascular density, and disease-specific survival was compared between the patient groups. Patients with FoxP3 positive cells within CD31-high tumors had significantly better survival than patients with FoxP3 positive cells in CD31-low tumors [HR: 2.314 (95% CI 1.049–5.106); *p* = 0.0377] (Figure 6A). Patients with FoxP3 positive cells in non-vascularized tumors had similar survival to patients without any FoxP3 infiltrates [HR: 1.013 (95% CI 0.5464–1.879); *p* = 0.9669] (Figure 6A). Thus, FoxP3-positive TIL are associated with improved outcome only when they are present in well-vascularized tumors.

Patients showed a moderate improvement in survival when their tumors expressed high levels of CD31 and contained TIA-1 positive cells (*p* = 0.0601, Figure 6B) or CD8 T cells (*p* = 0.0818, Figure 6C) compared to patients with expression of these markers in CD31-low tumors. Patients with high CD31 expressing tumors showed a modest improvement in survival when their tumors contained CD4 T cells (*p* = 0.1752, Figure S2A) and granzyme B expressing cells (*p* = 0.1785, Figure S2B) compared to patients with expression of these markers in CD31-low tumors.

Additionally, patients had significantly improved survival when their tumors expressed high levels of VEGF and contained granzyme B positive cells compared to patients with tumors expressing low levels of VEGF and containing granzyme B positive cells [HR: 2.522 (95% CI 1.097–5.799); *p* = 0.0294] (Figure 6D). Patients with granzyme B expression in non-vascularized tumors had similar survival outcomes compared to those patients lacking granzyme B positive cells in their tumors (*p* = 0.2973, Figure 6D), indicating granzyme B expression may not be beneficial in non-vascularized tumors. Patients with CD8 T cells in VEGF-high expressing tumors showed a moderate improvement in survival (*p* = 0.0876, Figure 6E) compared to patients with immune cells in low VEGF expressing tumors. Little significance was observed when patients had CD4 (*p* = 0.2683, Figure S2C), FoxP3 (*p* = 0.4448, Figure S2D) or TIA-1 (*p* = 0.2349, Figure S2E) expressing cells in VEGF-high tumors compared to patients with tumors containing these immune markers in VEGF-low tumors.

## Discussion

TIL can promote positive survival outcomes in high-grade serous ovarian cancer [4], [5], [28], [29], however, as we have shown, this can be dependent on the tumor environment. We sought to determine whether vascular density, an indicator of the oxygen level within tumors, would have an impact on the prognostic value of TIL based on the survival of high-grade serous ovarian carcinoma patients. Our results show that patients with highly vascularized tumors positive for markers of immune infiltrates and immune function have improved survival compared to patients with immune markers in tumors with low levels of vascularity. Further, our *in vitro* data suggests T cell function is drastically impaired under hypoxic conditions.

We found that high-grade serous ovarian cancer patients with high CD31 staining in their tumors had improved disease-specific survival compared to patients with low CD31 staining tumors. Reports on the formation of tumor vasculature as an independent prognostic indicator in ovarian carcinomas have been conflicted: having been shown to correlate with poor survival [30] and having no impact [31]. One explanation for these contrasting reports may be due to inclusion of multiple ovarian carcinoma types, confounding analysis in these studies. We found that neither clear cell nor endometrioid types had improved survival with high CD31 staining (Table S2 and Figure S3).

The benefits of TIL have been previously reported in multiple studies of ovarian cancer [4], [5], [28], [29]; we therefore speculate these results may also be dependent on the tumor vasculature. Given our *in vitro* data, the presence of TIL in tumors that are well-vascularized may lead to an optimal anti-tumor reaction whereas tumor hypoxia would suppress TIL function leading to poor outcomes. Additionally, increased vasculature may allow for the improved delivery of chemotherapy drugs. While the tumor specimens used in this study were collected prior to chemotherapy and the vascularity may have been modified, given that high-grade serous carcinoma is initially more responsive to chemotherapy than other subtypes [1], this combination of factors appears likely to be influencing improved patient survival. Increased vasculature in tumors may also promote infiltration of other immune cell types such as macrophages, myeloid-derived suppressor cells (MDSCs) and Th17 cells. The balance of these various cell types will ultimately promote or inhibit the anti-tumor response. For example, M2 macrophages and MDSCs can impair the TIL response [32]–[34], while Th17 cells have been shown to promote anti-tumor activity [35]. However, there are conflicting reports in the literature describing Th17 cells as having both pro- and anti-tumor functions [36].

Hypoxia appears to profoundly influence T cell differentiation and function. Our data demonstrate that hypoxia causes a reduction in IFNγ production, findings which are supported by Lukashev *et al.* [37]. Hypoxia also reduces CD8 T cell-mediated cytolytic activity, which is consistent with previous findings by Noman *et al.* [16]. This would imply that, *in vivo*, tumor hypoxia would have a similar effect on TIL function. While our *in vivo* studies showed that patients with ovarian tumors positive for the cytotoxic molecules TIA-1 and granzyme B in CD31 and VEGF-low tumors had decreased survival compared to those with cytotoxic molecules expressed in CD31 and VEGF-high tumors, these observations were not as drastic as those observed *in vitro*. This may be due to the highly controlled nature of our *in vitro* experiments, which consisted solely of oxygen deprivation. For example, our *in vitro* experiments are based on the assumption that hypoxia *in vivo* is a constant event occurring at 1.5% oxygen, however, the *in vivo* setting likely includes additional dynamic interactions with many cell types and various nutrient conditions, which can strongly influence the ability of a T cell to function. This discordance may also result due to practical challenges of serial immunohistochemistry staining, whereby the geography, orientation and tumor architecture in relationship to hypoxia may have changed during each subsequent tissue section. Finally, tissue-microarrays (TMAs) unintentionally introduce tissue bias due to the selection of the core. Most cores used in TMAs are selected based on a defined degree of tumor versus stroma versus epithelium and often exclude areas of necrosis where hypoxia is typically, although not always, observed. While many of these hurdles cannot be overcome with large retrospective TMAs, these factors may introduce variables that could be more or less representative of the type of controlled experiments *in vitro*. However, this suggests that more detailed studies are needed to evaluate the extent of the TIL and co-localization with markers of hypoxia in primary tumors. Within tumors, hypoxia may also indirectly cause decreased anti-tumor activity by decreasing antigen presentation by macrophages and dendritic cells. Indeed, hypoxia has been shown to decrease phagocytosis and inhibit migration in macrophages [38], and may cause dendritic cells to promote type 2 polarization in T helper cells, thus impairing cell-mediated immunity [39].

While hypoxia had a dramatic effect on T cell function *in vitro*, during this time T cells activated autophagy. Hypoxia-induced autophagy is a well-established mechanism for tumor cell survival [12], [40]. However, to our knowledge, this is the first study to demonstrate that hypoxia activates autophagy in CD8 T cells. Hypoxia-induced autophagy may be important for the production of metabolites to support T cell growth and for the clearance of mitochondria that produce reactive oxygen species during hypoxia [41]. Both of these functions of autophagy could be important for maintaining viability within the tumor environment or until an adequate vasculature is re-established and capable of supporting continued T cell growth, proliferation and anti-tumor function.

Finally, we assessed the importance of vascularity in combination with immune infiltrates and markers of immune function on patient outcomes. Vascularity was chosen as an indicator of tumor oxygenation because animal models have established that hypoxia occurs as a consequence of inadequate vasculature [21], [22]. Interestingly, we found that FoxP3 positive infiltrates were beneficial for patient survival in CD31-high expressing tumors compared to FoxP3 positive infiltrates in CD31-low expressing tumors. FoxP3 is a well-recognized marker of T reg cells [8], and contrary to some reports, FoxP3 expressing cells have been associated with favorable patient survival [5], [9], while T regs have been associated with negative patient outcomes in ovarian carcinoma due to their role in actively suppressing T cells [6], [7]. Emerging evidence suggests that the reason for this discrepancy is due to the fact that FoxP3 is not an exclusive marker expressed on immune suppressing T regs. In this context, FoxP3 positive cells may represent activated cytolytic lymphocytes, which could explain their association with good outcome [42]. Additionally, given the plasticity of T regs, the FoxP3 positive cells observed in vascularized tumors may also have the potential to represent a partially or fully differentiated Th17 cell. In turn, Th17 cells can synergize with, and acquire a protective Th1 response [35], [36]. Increased levels of FoxP3 may also indicate that cytolytic T cells are also present at increased numbers within the tumor [43]. To address these possibilities, future studies will require prospective isolation of TIL and assessment of T reg suppression activity using multi-parameter staining.

A report by Facciabene *et al.* using an epithelial murine ovarian cancer model showed that hypoxic tumors recruit FoxP3 expressing T reg cells to the tumor and these cells suppress anti-tumor immune cell subsets [15]. However, we did not observe a higher number of FoxP3 positive T cells in non-vascularized tumors compared to vascularized tumors (Table S3). Thus, a higher percentage of FoxP3 positive T cells may not account for the decrease in patient survival observed when FoxP3 expressing cells are located in non-vascularized environments. One report regarding ovarian carcinoma found that a high ratio of CD8 T cells to T regs was positively associated with improved patient survival [29]. We were not able to assess the ratio of FoxP3 to CD8 cells in our cohort given the scoring methods used for CD8. This may account for the differences in survival observed when patients' tumors contained FoxP3 expressing cells in vascularized versus non-vascularized environments.

Given that a significant number of high-grade serous ovarian carcinomas show immune cell infiltration [5], and that this infiltration is positively associated with patient survival, we sought to determine how the ovarian tumor environment impacts immune cell function. We found that patients generally had worse survival outcomes when immune infiltrates were in a non-vascularized environment as compared to a vascularized environment. One important consideration in this study is the use of CD31 and VEGF as proxies for hypoxia. Indeed, the expression of other markers such as HIF-1α or its targets, including carbonic anhydrase IX, have also been used to demarcate a hypoxic environment. While these are all reasonable markers, some represent early or acute hypoxia [44] while others may not solely be expressed in hypoxic regions [19]. This may in part explain why some immune markers strongly associated with outcomes, while others exhibited more subtle trends. Understanding how T cells function under conditions such as low oxygen remains a major barrier in improving tumor specific T cell immunity. However, even with the most effective new immunotherapeutic strategies, we will require knowledge on how to reprogram T cells to adapt or overcome this metabolically suppressive state.

## Materials and Methods

### Ethics statement

All individuals were identified through the Cheryl Brown Ovarian Cancer Outcomes Unit ovarian cancer registry and approval for curation of the specimens as well as acquisition of clinical outcome information was obtained from the Research Ethics Board of the University of British Columbia, BC Cancer Agency Research Ethics Board (H02-61375). Patient anonymity was preserved for all specimens through the use of Ethics Board approved case coding. Mouse studies were approved by the University of Victoria Animal Care Committee (Animal Care Protocol #2011-009(3)).

### Patient population

The retrospective patient cohort used for this study has been described previously [45]. Briefly, the Cheryl Brown Ovarian Cancer Outcomes Unit collected ovarian tumor specimens from 1984–2000 from women located in British Columbia. Patients were treated with surgery resulting in no macroscopic residual disease and had platinum-based chemotherapy. Tumors were collected at primary surgery prior to treatment with chemotherapy, fixed with 10% neutral-buffered formalin, processed and embedded in paraffin. A representative area of each tumor was selected and 0.6 mm cores in duplicate were used to construct a TMA. The follow-up data used for our analysis had a median of 5.3 years. For Kaplan-Meier analysis, overall survival was defined as survival without death due to any cause, progression-free survival was survival without evidence of disease recurrence and disease-specific survival was survival without death due to ovarian cancer.

### Immunohistochemistry marker scoring and analysis

The ovarian tumor TMA was stained for the following markers: CD8, CD4, granzyme B [4], FoxP3, TIA-1 [5], CD31 and VEGF. Immune marker scores were binarized as positive or negative in the epithelium. VEGF scores were binarized into ‘high’ and ‘low’ categorizes based on a threshold of 50% positive staining. CD31 vascular density scores were separated into groups of ‘high’ and ‘low’ based on the 33rd percentile. CD8, CD4 and granzyme B were scored as described by Clarke *et al.* [4] according to the following criteria: cores containing no positive cells were scored as 0, cores containing positive staining cells within the stroma were scored as 1, cores containing positive staining cells in the epithelium were scored as 2 and cores containing positive cells in both the epithelium and stroma were scored as 3. The scores were then binarized and patients were separated into two groups for analyses where patients were considered positive for the respective marker if the tumor cores had immunohistochemistry scores of 2 or 3, and were considered negative if the tumor cores had immunohistochemistry scores of 0 or 1. TIA-1 and FoxP3 scores were assigned as described by Milne *et al.* [5] based on cells located within the tumor epithelium only as follows: 0 (no cells), 1 (1–5 cells), 2 (6–19 cells), or 3 (≥20 cells). Results were binarized into groups of positive (immunohistochemistry score 1, 2, 3) or negative (immunohistochemistry score 0). VEGF was scored as 0 for tumors which had no staining, 1 for tumors which had 1–50% positive tumor cells and 2 for tumors which had greater than 50% positive staining. CD31 was scored at 100× magnification using a Chalkley grid consisting of 8 horizontal and 6 vertical cross hairs. Each core was scored for the number of cross hairs which intersected positively staining vasculature (V), tumor epithelium (T), or stroma (S). Samples were then assigned a vascular density score. This was assigned by first determining the proportion of stroma (PS) in each core by the formula (S+V)/(S+V+T) = PS. Vascular density was then calculated using the formula V/PS. The highest vascular density score within a set of duplicate cores was used for our analysis.

### Immunohistochemistry staining

CD8, CD4 and granzyme B staining was performed by Clarke *et al.* [4] using the following antibodies specific for CD8 (Dako, Burlington, ON, CA; clone C8/144B, mouse monoclonal, 1∶50), CD4 (Novocastra, Concord, ON, CA; clone 4B12, mouse monoclonal, 1∶50) and granzyme B (Dako; clone GrB-7, mouse monoclonal, 1∶25). TIA-1 and FoxP3 staining was performed by Milne *et al.* [5] using the following antibodies specific for TIA-1 (Abcam, Cambridge, MA, USA; clone TIA-1, mouse monoclonal, 1∶50), and FoxP3 (eBioscience, San Diego, CA, USA; clone Ebio7979, mouse monoclonal, 1∶50). VEGF staining was carried out with steaming for 40 minutes in Dako Target Retrieval solution. The primary antibody anti-VEGF (R&D Systems, Minneapolis, MN USA; clone 26503, mouse monoclonal) was used at a 1∶50 dilution for 20 minutes at room temperature. CD31 staining was carried out as described by Milne *et al.* [5]. Briefly, tumor tissues were sectioned at 4 µm onto Superfrost plus slides (Fisher Scientific, Nepean, ON, CA) and incubated overnight at 37°C. The slides were deparaffinized in xylene and graded alcohols and a Ventana Discovery XT autostainer (Ventana, Tucson, AZ, USA) was used for immunohistochemistry staining. Ventana's standard CC1 protocol was used for antigen retrieval. The primary antibody anti-CD31 (Cell Marque, Rocklin, CA, USA; clone JC70, mouse monoclonal) was added to the slides at a dilution of 1∶100 in Ventana's Antibody Diluent and incubated for 60 minutes. A cross-adsorbed biotinylated goat anti-mouse IgG secondary antibody (Jackson Immunoresearch, West Grove, PA, USA) was manually applied at a dilution of 1∶250 for 32 minutes. Bound antibodies were detected using the DABMap kit (Ventana), counterstained with hematoxylin (Ventana), and coverslipped manually with Cytoseal-60 (Richard Allan, Kalamazoo, MI, USA).

### Cell culture and hypoxic conditions

EL4 cells, a thymoma tumor cell line and its derivative cell line E.G7, which has been transfected with an ovalbumin (OVA) and geneticin resistance expressing construct were purchased from ATCC (Manassas, VA, USA). Primary mouse T cells, EL4 and E.G7 cells, were cultured in cRP10 media consisting of 1640 RPMI media (Fisher Scientific) containing the following supplements: 1 mM sodium pyruvate, 1 mM Hepes, 50 U/ml penicillin, 50 µg/ml streptomycin, 1 mM L-glutamine, 10% fetal bovine serum (all from Fisher Scientific), and 50 µM 2-mercaptoethanol (Sigma-Aldrich, Oakville, ON, CA). For E.G7 cell culture, cRP10 media was supplemented with 400 µg/ml G418 (Fisher Scientific) and glucose (Sigma-Aldrich) to reach a final concentration of 4.5 mg/ml glucose in the media. For all hypoxia experiments, cells were placed in a humidified hypoxia chamber (Coy Laboratories, Grass Lake, MI, USA) at 37°C with 1.5% oxygen, 5% CO_2_, and 93.5% nitrogen and cultured for varying time periods as indicated.

### Mice

OT-I transgenic mice were purchased from Jackson Laboratories (Bar Harbor, ME, USA).

### T cell activation

Following isolation from harvested mouse spleens, OT-I expressing splenocytes were activated with 2 µg/ml SIINFEKL peptide (Anaspec, Fremont, CA, USA) for 2 hours prior to removal of the media and replenishment with fresh cRP10 media. For cytotoxicity assays, OT-I splenocytes were activated in a 10∶1 ratio with irradiated (100 Gy, Varian Clinac 6EX medical linear accelerator) EL4 cells pulsed with 0.5 µg SIINFEKL peptide per 1 million cells. 100 U/ml interleukin-2 (Peprotech, Dollard des Ormeaux, QC, CA) was added on day 1 of culture and added to the media with every passage following.

### Autophagic flux and Western blotting

To monitor autophagy induction, T cells were cultured under 1.5% oxygen for 20 hours and treated with or without 50 µM chloroquine (CQ) (Sigma-Aldrich). As a positive control for HIF-1α protein detection, cells were treated with cobalt chloride at 100 µM (Sigma-Aldrich). Cell pellets were lysed and boiled at 99°C for 10 minutes with shaking on a thermomixer at 1500 rpm (Eppendorf, Mississauga, ON, CA). Protein lysates were run on 4–12% Bis-Tris or 3–8% Tris-Acetate polyacrylamide gels (Invitrogen, Burlington, ON, CA), transferred onto a nitrocellulose membrane (Life Sciences, Pensacola, FL, USA). Blots were probed with the following primary antibodies at the specified concentrations overnight: anti-Atg5 at 1∶1000 (Novus Biologicals, Oakville, ON, CA; rabbit polyclonal), anti-HIF-1α at 1∶1000 (Cayman, Ann Arbor, MI, USA; rabbit polyclonal), anti-LC3 at 1∶2000 (MBL, Des Plaines, IL, USA; rabbit polyclonal), anti-p62 at 1∶2000 (Sigma-Aldrich; rabbit polyclonal) and anti-β-actin at 1∶10,000 (Sigma-Aldrich; clone AC-15, mouse monoclonal). Secondary antibodies including goat anti-rabbit IgG (H&L) IRDye®800 conjugated (Rockland, Gilbertsville, PA, USA) and Alexa Fluor 680 goat anti-mouse IgG (Invitrogen) were used at a 1∶10,000 dilution. Immunoblots were quantified using the Odyssey program version 3.0 (LI-COR, Lincoln, NE, USA). All quantified protein band intensities were normalized to β-actin. For p62 quantification during hypoxia, the fold change below normoxia was determined. The fold change over normoxia plus CQ treatment was determined for LC3 quantification during hypoxia.

### Cytotoxicity assay

Splenocytes were activated and cultured for 6 days. The cells were then primed under 1.5% oxygen or 21% oxygen for 16 hours. EL4 and E.G7 cells were labeled with 0.75 µM carboxyfluorescein succinimidyl ester (CFSE, Sigma-Aldrich) and co-cultured with CD8 T cells at the indicated ratios for 4 hours under normal conditions or 1.5% oxygen. 7-amino-actinomycin D (7-AAD, Sigma-Aldrich) was added to the cells at 10 µg/ml prior to cell flow cytometric analysis by Guava EasyCyte (Millipore, Billerica, MA, USA). Acquisition and analysis was completed using the cytosoft 5.3 cell toxicity program (Guava Technologies Inc. Hayward, CA, USA). Percent killing was corrected using the formula ((sample death-spontaneous death)/(maximum death-spontaneous death)) * 100%. Maximal death was determined by heat killing tumor target cells for a minimum of 1 hour at 55°C. Spontaneous death was determined by the amount of death in tumor target cultures alone.

### Intracellular cytokine staining

Splenocytes were activated and cultured for 6 days. The cells were then primed under 1.5% oxygen or normoxia for 15 hours. On day 7, the cells were restimulated using 10 ng/ml phorbol myristate acetate (PMA) and 0.5 µg/ml ionomycin (both Sigma-Aldrich) in the presence of 4 µl/6 ml golgistop (BD Biosciences, Mississauga, ON, CA) and 5 µg/ml brefeldin A (Sigma-Aldrich) for 5 hours under 1.5% oxygen or under normal conditions. Intracellular cytokines were detected using a FoxP3 intracellular staining kit (eBioscience) following the manufacturer's instructions. Cells were stained with anti-CD8 allophycocyanin-H7 (APC H7, BD Bioscience; clone 53-6.7, rat monoclonal) at 1∶200. The following antibodies were used at the indicated concentrations for intracellular staining: anti-IFNγ phyco-erythrin (PE, BD Biosciences; clone XMG1.2, rat monoclonal) at 1∶100 and anti-TNFα fluorescein isothiocyanate (FITC, eBioscience; clone MP6-XT22, rat monoclonal) at 1∶100. Flow cytometry was performed with a FACSCalibur flow cytometer (BD Biosciences). Data analysis was completed using FlowJo 7.6.5 software (Tree Star, Ashland, OR, USA). Mean fluorescence intensities were assessed by the geometric means.

### Statistics

Statistical analysis was carried out using GraphPad Prism software version 5.02 (GraphPad Software, La Jolla, CA, USA). Statistically significant *p* values were considered to be those less than 0.05. Based on the D'Agostino and Pearson omnibus normality test results for CD31 (*p*<0.0001), Mann-Whitney rank-sum tests were used to compare differences between the CD31 vascular density scores observed for patients based on age (split on the median), stage and those positive or negative for immune cell markers. Fisher's exact tests were used to carry out contingency analyses. A one-sample t-test on log transformed values compared to a hypothetical mean of zero was used to assess the significance of protein expression. Paired t-tests were used to compare significant differences between the mean values observed for independent intracellular staining experiments. Based on the D'Agostino and Pearson omnibus normality test results for the cytotoxicity assay (*p* = 0.0356), the values were log transformed and paired t-tests were used to compare significant differences between the average of the mean values observed under hypoxia and normoxia. Kaplan-Meier curves were compared using log-rank tests.

## Supporting Information

Figure S1 **High-grade serous ovarian tumors express the vasculature marker CD31.** Kaplan-Meier analysis of (A) overall survival and (B) progression-free survival in high-grade serous ovarian cancer patients. Statistical significance was assessed using a Log-rank test.
(DOCX)

Figure S2 **Vascularized tumors containing TIL and functional markers show a modest association with improved patient survival.** (A) CD31 and CD4, (B) CD31 and Granzyme B (GzmB), (C) VEGF and CD4, (D) VEGF and FoxP3, (E) VEGF and TIA-1. The indicated *p* values were determined using a Log-rank test.
(DOCX)

Figure S3 **Clear cell and endometrioid carcinomas express the vasculature marker CD31.** Kaplan-Meier analysis of disease-specific survival for (A) clear cell carcinoma and (B) endometrioid carcinoma patients. Statistical significance was assessed using a Log-rank test.
(DOCX)

Table S1 **Contingency analysis.** The number of patients in each indicated category were compared: CD31-low, CD31-high or VEGF-low, VEGF-high and infiltrate positive or negative. Statistical significance was assessed using a Fisher's exact test.
(DOCX)

Table S2 **Patient characteristics, follow-up time and survival characteristics for endometrioid carcinoma and clear cell ovarian carcinoma cases.**
(DOCX)

Table S3 **FoxP3 scores in high-grade serous ovarian carcinoma separated into CD31-high and CD31-low.** The FoxP3 scores in CD31-high and CD31-low tumors are indicated. FoxP3 cells in the epithelium were counted as follows: 0 (no cells), 1 (1–5 cells), 2 (6–19 cells), or 3 (≥20 cells).
(DOCX)
