# Supplementary material for: Markers of T Cell Infiltration and Function Associate with Favorable Outcome in Vascularized High-Grade Serous Ovarian Carcinoma
Source: PLoS One. 2013 Dec 23;8(12):e82406. doi: 10.1371/journal.pone.0082406 (PMC3871161; doi:10.1371/journal.pone.0082406)
Supplement: Figure S1 — High-grade serous ovarian tumors express the vasculature marker CD31. Kaplan-Meier analysis of (A) overall survival and (B) progression-free survival in high-grade serous ovarian cancer patients. Statistical significance was assessed using a Log-rank test. (DOCX) [file pone.0082406.s001.docx]

**B**

**A**

**Figure S1. High-grade serous ovarian tumors express the vasculature marker CD31.** Kaplan-Meier analysis of (A) overall survival and (B) progression-free survival in high-grade serous ovarian cancer patients. Statistical significance was assessed using a Log-rank test.
