# Supplementary material for: Markers of T Cell Infiltration and Function Associate with Favorable Outcome in Vascularized High-Grade Serous Ovarian Carcinoma
Source: PLoS One. 2013 Dec 23;8(12):e82406. doi: 10.1371/journal.pone.0082406 (PMC3871161; doi:10.1371/journal.pone.0082406)
Supplement: Figure S2 — Vascularized tumors containing TIL and functional markers show a modest association with improved patient survival. (A) CD31 and CD4, (B) CD31 and Granzyme B (GzmB), (C) VEGF and CD4, (D) VEGF and FoxP3, (E) VEGF and TIA-1. The indicated p values were determined using a Log-rank test. (DOCX) [file pone.0082406.s002.docx]

**C**

**A**

**B**

**D**

**E**

**Figure S2.** **Vascularized tumors containing TIL and functional markers show a modest association with improved patient survival.** (A) CD31 and CD4, (B) CD31 and Granzyme B (GzmB), (C) VEGF and CD4, (D) VEGF and FoxP3, (E) VEGF and TIA-1. The indicated *p* values were determined using a Log-rank test.
