# Supplementary material for: Markers of T Cell Infiltration and Function Associate with Favorable Outcome in Vascularized High-Grade Serous Ovarian Carcinoma
Source: PLoS One. 2013 Dec 23;8(12):e82406. doi: 10.1371/journal.pone.0082406 (PMC3871161; doi:10.1371/journal.pone.0082406)
Supplement: Figure S3 — Clear cell and endometrioid carcinomas express the vasculature marker CD31. Kaplan-Meier analysis of disease-specific survival for (A) clear cell carcinoma and (B) endometrioid carcinoma patients. Statistical significance was assessed using a Log-rank test. (DOCX) [file pone.0082406.s003.docx]

**Figure S3. Clear cell and endometrioid carcinomas express the vasculature marker CD31.** Kaplan-Meier analysis of disease-specific survival for (A) clear cell carcinoma and (B) endometrioid carcinoma patients. Statistical significance was assessed using a Log-rank test.
