# Supplementary material for: Markers of T Cell Infiltration and Function Associate with Favorable Outcome in Vascularized High-Grade Serous Ovarian Carcinoma
Source: PLoS One. 2013 Dec 23;8(12):e82406. doi: 10.1371/journal.pone.0082406 (PMC3871161; doi:10.1371/journal.pone.0082406)
Supplement: Table S1 — Contingency analysis. The number of patients in each indicated category were compared: CD31-low, CD31-high or VEGF-low, VEGF-high and infiltrate positive or negative. Statistical significance was assessed using a Fisher's exact test. (DOCX) [file pone.0082406.s004.docx]

|  | **CD31** | | **VEGF** | |
| --- | --- | --- | --- | --- |
|  | **low** | **high** | **low** | **high** |
| **CD8 -** | 21 | 19 | 35 | 6 |
| **CD8 +** | 40 | 111 | 111 | 37 |
| *p* value | 0.0037 | | 0.2078 | |
| **CD4 -** | 39 | 66 | 90 | 13 |
| **CD4 +** | 21 | 64 | 54 | 30 |
| p value | 0.0842 | | 0.0002 | |
| **FoxP3 -** | 35 | 52 | 74 | 12 |
| **FoxP3 +** | 24 | 75 | 67 | 31 |
| *p* value | 0.0267 | | 0.0052 | |
| **Granzyme B -** | 42 | 68 | 90 | 19 |
| **Granzyme B +** | 19 | 61 | 56 | 24 |
| p value | 0.0412 | | 0.0533 | |
| **TIA-1 -** | 28 | 34 | 52 | 11 |
| **TIA-1 +** | 32 | 94 | 91 | 32 |
| *p* value | 0.0079 | | 0.2047 | |

**Table S1**. **Contingency analysis.** The number of patients in each indicated category were compared: CD31-low, CD31-high or VEGF-low, VEGF-high and infiltrate positive or negative. Statistical significance was assessed using a Fisher’s exact test.
