# Supplementary material for: Markers of T Cell Infiltration and Function Associate with Favorable Outcome in Vascularized High-Grade Serous Ovarian Carcinoma
Source: PLoS One. 2013 Dec 23;8(12):e82406. doi: 10.1371/journal.pone.0082406 (PMC3871161; doi:10.1371/journal.pone.0082406)
Supplement: Table S2 — Patient characteristics, follow-up time and survival characteristics for endometrioid carcinoma and clear cell ovarian carcinoma cases. (DOCX) [file pone.0082406.s005.docx]

|  | **Endometrioid** | **Clear cell** |
| --- | --- | --- |
| **Age at surgery (years)** | | |
| Median | 53.7 | 54.7 |
| Range | 29.45 - 88.07 | 28.1 - 89.1 |
| **Silverberg grade** | | |
| 1 | 80 (65.0%) | 0 (0%) |
| 2 | 35 (28.5%) | 0 (0%) |
| 3 | 8 (6.5%) | 130 (100%) |
| Not graded | 0 (0%) | 0 (0%) |
| **FIGO stage^1^** | | |
| I | 69 (56.1%) | 66 (50.8%) |
| II | 48 (39.0%) | 56 (43.1%) |
| III | 6 (4.9%) | 8 (6.2%) |
| Total number patients | 123 | 130 |
| **Follow up time** | | |
| Median follow-up (range), years | 7.90 (0.1 - 21.2) | 6.4 (0.3 - 23.6) |
| Survival characteristics | | |
| Disease progressions | 21 (17.1%) | 44 (33.8%) |
| Ovarian cancer deaths | 20 (16.3%) | 40 (30.8%) |
| Total number of deaths | 40 (32.5%) | 56 (43.1%) |

**Table S2.** **Patient characteristics, follow-up time and survival characteristics for endometrioid carcinoma and clear cell ovarian carcinoma cases.**

^1^FIGO=Federation of Gynecology and Obstetrics.
