# Supplementary material for: Markers of T Cell Infiltration and Function Associate with Favorable Outcome in Vascularized High-Grade Serous Ovarian Carcinoma
Source: PLoS One. 2013 Dec 23;8(12):e82406. doi: 10.1371/journal.pone.0082406 (PMC3871161; doi:10.1371/journal.pone.0082406)
Supplement: Table S3 — FoxP3 scores in high-grade serous ovarian carcinoma separated into CD31-high and CD31-low. The FoxP3 scores in CD31-high and CD31-low tumors are indicated. FoxP3 cells in the epithelium were counted as follows: 0 (no cells), 1 (1–5 cells), 2 (6–19 cells), or 3 (≥20 cells). (DOCX) [file pone.0082406.s006.docx]

| **FoxP3 scores in CD31-high tumors** | | | | |
| --- | --- | --- | --- | --- |
| **Foxp3 score** | **0** | **1** | **2** | **3** |
| **Number of tumors** | 25 | 50 | 44 | 9 |
| **Percent of CD31-high (%)** | 19.53 | 39.06 | 34.38 | 7.03 |
| **FoxP3 scores in CD31-low tumors** | | | | |
| **Foxp3 score** | **0** | **1** | **2** | **3** |
| **Number of tumors** | 30 | 16 | 16 | 0 |
| **Percent of CD31-high (%)** | 48.39 | 25.81 | 25.81 | 0 |

**Table S3**. **FoxP3 scores in high-grade serous ovarian carcinoma separated into CD31-high and CD31-low.** The FoxP3 scores in CD31-high and CD31-low tumors are indicated. FoxP3 cells in the epithelium were counted as follows: 0 (no cells), 1 (1-5 cells), 2 (6-19 cells), or 3 (≥20 cells).
